# Illustrations of the Inductive Method in Medicine

**Published:** 1892-12

**Authors:** 


*Illustrations of the Inductive Method in Medicine.* By William Murray, M.D., F.R.C.P. Pp. 158. London: H. K. Lewis. 1891.

This little book consists of a series of essays written in a clear and pleasant style by a man who has had a large practical experience, and has thought over and digested the problems which must arise in the mind of every thoughtful man in the daily practice of our profession. Certainly in medicine and its allied sciences we are at present liable to be overwhelmed with the wealth of detail constantly accumulated by the ceaseless activity of the host of workers, and no need is more pressing than that some master mind should arise and, in a philosophy of medicine, reduce to order the particular cases, by setting forth the general principles by which they are governed. Whilst agreeing with the author, that if medicine is to advance as a science, it must make progress by a constant effort to arrive at these general principles, we would yet emphasise the necessity, more evident perhaps in medicine than in any other science, of making sure that our premisses are wide enough to justify us in laying down a general rule. The tendency has always been for medical men to be too ready to lay down absolute laws from insufficient clinical data, and thus to retard real progress. The other essays in the book are meant to illustrate the manner in which the inductive method may be safely employed. They are original in matter, and are chiefly concerned with problems of digestion, of elimination, and of therapeutics.

